# Variation on Molecular Structure, Crystallinity, and Optical Properties of Dentin Due to Nd:YAG Laser and Fluoride Aimed at Tooth Erosion Prevention

**DOI:** 10.3390/ijms19020433

**Published:** 2018-02-01

**Authors:** Daísa L. Pereira, Anderson Z. Freitas, Luciano Bachmann, Carolina Benetti, Denise M. Zezell, Patricia A. Ana

**Affiliations:** 1 Center for Engineering, Modeling and Applied Social Sciences, Universidade Federal do ABC, Sao Bernardo do Campo, SP 09606-045, Brazil; daisa@usp.br; 2 Center for Lasers and Applications, Instituto de Pesquisas Energéticas e Nucleares, IPEN-CNEN/SP, Sao Paulo, SP 05508-000, Brazil; freitas.az@ipen.br; 3 Faculdade de Filosofia, Ciências e Letras de Ribeirão Preto, Universidade de Sao Paulo, Ribeirao Preto, SP 14040-900, Brazil; bachmann@ffclrp.usp.br; 4 Center for Engineering, Modeling and Applied Social Sciences, Universidade Federal do ABC, Sao Bernardo do Campo, SP 09606-045, Brazil; c.benetti@ufabc.edu.br; 5 Center for Lasers and Applications, Instituto de Pesquisas Energéticas e Nucleares, IPEN-CNEN/SP, Sao Paulo, SP 05508-000, Brazil; 6 Center for Engineering, Modeling and Applied Social Sciences, Universidade Federal do ABC, Sao Bernardo do Campo, SP 09606-045, Brazil; patricia.ana@ufabc.edu.br

**Keywords:** dentin, infrared laser, composition, erosion, crystallinity, fluoride, abrasion

## Abstract

This in vitro study evaluated the compositional, crystalline, and morphological effects promoted by Nd:YAG laser on root dentin, and verified the effects of laser and topical acidulated phosphate fluoride application (APF-gel) on dentin erosion. 180 bovine dentin slabs were randomized into 4 groups (*n* = 45): G1–untreated, G2–APF-gel (1.23% F^−^, 4 min), G3–Nd:YAG (1064 nm, 84.9 J/cm^2^, 10 Hz), and G4–APF-gel application followed by Nd:YAG laser irradiation. The compositional, crystalline, and morphological effects promoted by treatments were investigated on five samples of each experimental group. The other samples were submitted to a 5-day, 10-day, or 15-day erosive and abrasive demineralization and remineralization cycling in order to create erosion lesions. The area and depth of lesions, as well as the optical attenuation coefficient, were assessed, and all data were statistically analysed (*p* < 0.05). Nd:YAG laser promoted the reduction of carbonate, the formation of tetracalcium phosphate, as well as the melting and recrystallization of the dentin surface. Laser significantly decreased the area and depth of erosion lesions and altered the optical attenuation coefficient when compared to untreated and APF-gel groups, but the association of APF-gel and laser did not promote an additional effect. Nd:YAG laser irradiation can be a promissory treatment to prevent dentin erosion and the abrasion process.

## 1. Introduction

Dental erosion is an oral demineralization process whose prevalence has increased significantly [1,2,3,4]. In a simplified form, it is an irreversible loss of enamel and/or dentin caused by extrinsic or intrinsic acids in the absence of biofilm [5]. The etiology of the wear promoted by the erosive process is multifactorial, and the compliance of the patient is an essential aspect to be considered for preventing the development and progression of erosion lesions [6].

The use of high-intensity lasers has been proposed for preventing the appearance or progression of these lesions, since the literature reports promissory results for preventing dental caries using such technology [7,8,9]. Depending on laser parameters such as wavelength, energy density, pulse width, and repetition rate, high-intensity infrared lasers can change the microstructure of enamel and dentin due to the localized heating and, thus, can decrease the solubility of these tissues to acid attack [10,11,12].

The Nd:YAG laser (1064 nm) has been frequently employed for caries preventive purposes, because successful results were reported from previous in vitro [12,13,14], in situ [15], and in vivo [5,15,16] studies. However, contradictory results are also observed, since it was reported that this laser wavelength cannot offer any additional benefit when compared to fluoride action singly [5,10]. On the other hand, previous studies performed with other laser wavelengths report some synergistic effect of laser and topical application of fluoride gel (APF-gel) on early caries development [6], which is related to the higher formation of calcium fluoride-like material (CaF_2_-like material) due to laser irradiation, which can extend the cariostatic effect of APF-gel. The Nd:YAG laser was also studied for treating the erosive process on dentin [17,18,19,20,21], and it was shown that laser changes the morphology of dentin, but these alterations were not enough to prevent or treat root erosion. However, no study employed a photoabsorber, which limits the effects of Nd:YAG laser irradiation, whose photons are poorly absorbed by dental hard tissues [7].

Due to these contradictory reports, and considering the popularity of Nd:YAG laser for caries-preventive purposes, it is necessary to know the chemical interactions of this laser, together with a photoabsorber and the fluoride in order to establish the real potential of the association of these strategies for preventing dentin erosion for a longer time. In this way, this in vitro study aimed to verify the chemical, crystallographic, morphological, and optical changes that occur on sound root dentin due to Nd:YAG laser irradiation, associated or not with APF-gel application, as well as to determine the influence of dentin treatment on the development and progression of erosion and abrasion lesions.

## 2. Results

**Figure 1:**
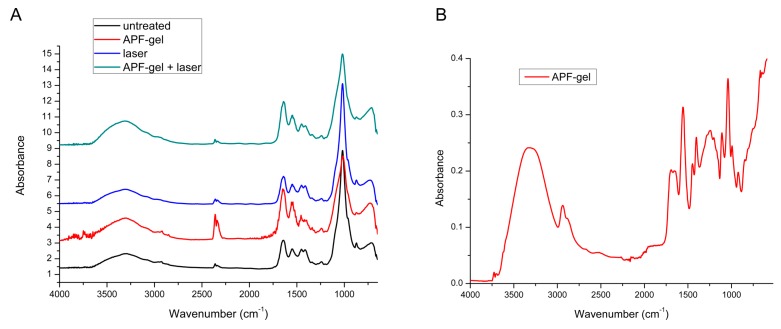
Means of infrared spectra in the range of 4000–650 cm^−1^: (**A**)—obtained after treatments proposed for dentin, evidencing the differences in intensity of bands analyzed according to the treatments; (**B**)—obtained from APF-gel used in this study.

**Figure 2:**
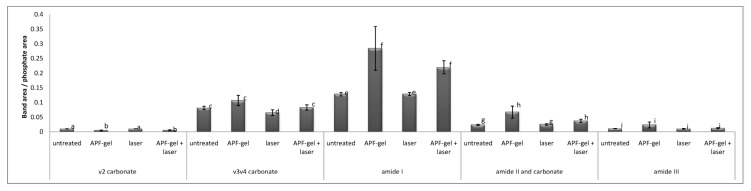
Means of ν_2_ carbonate/phosphate, ν_3_ν_4_ carbonate/phosphate, amide I/phosphate, amide II and carbonate/phosphate, and amide III/phosphate ratios obtained from dentin samples immediately after treatments. Bars denote standard errors. Distinct letters indicate statistically significant differences (*p* < 0.05) according to Kruskal-Wallis and Student-Newman-Keuls test.

The average of ATR-FTIR spectra of all treatment groups is shown in Figure 1, in which the main dentin infrared bands are evidenced. Note that none of the proposed treatments promoted the disappearance or appearance of new infrared bands; only a change in the intensity of the existing bands was observed. The semi-quantitative analysis of these bands is shown in Figure 2, and it is noticed that laser irradiation significantly reduced (*p* < 0.05) the proportion of ν_3_ν_4_ carbonate in relation to phosphate, while APF-gel increased significantly (*p* < 0.05) the content of ν_2_ carbonate, amide I, and amide II in relation to phosphate.

**Figure 3:**
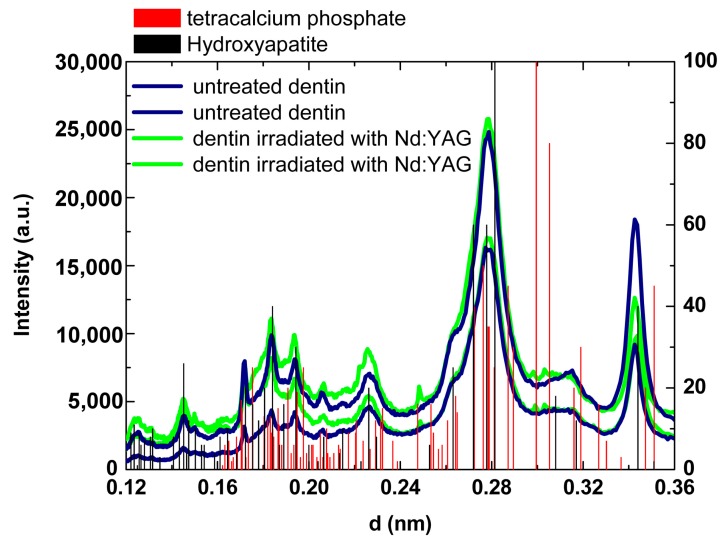
X-ray diffraction pattern of two samples of untreated and two samples of Nd:YAG laser irradiated dentin. The main Bragg peaks expected for hydroxyapatite (HAP; JCPDF 09-0432) and for tetracalcium phosphate (TetCP, JCPDF 25-1137) are indicated in black and red, respectively.

**Figure 4:**
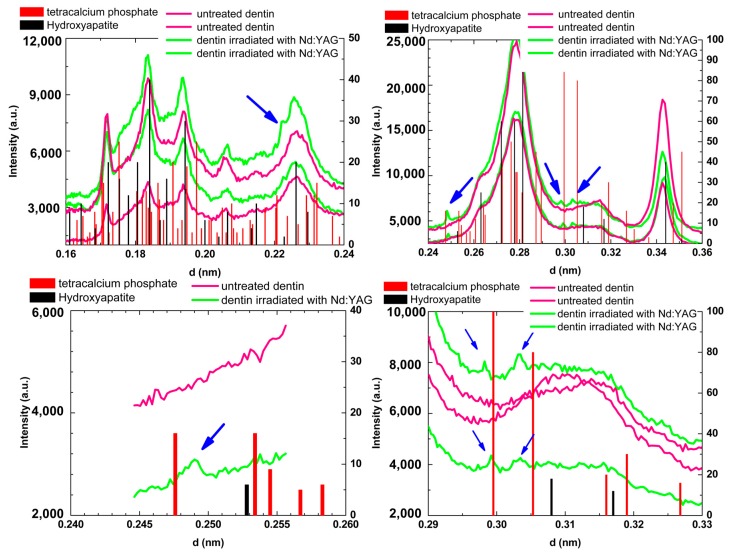
Experimental X-ray diffraction patterns of the untreated dentin, laser irradiated dentin samples, and the expected patterns for hydroxyapatite (HAP, JCPDF 09-0432) and for tetracalcium phosphate (TetCP, JCPDF 25-1137). The blue arrows indicate additional peaks on irradiated dentin that are close to peaks of TetCP.

In Figure 3, where d is the interplanar space, it is evidenced that the comparison of hydroxyapatite (JCPDF 09-0432) and tetracalcium phosphate (TetCP, JCPDF 25-1137) Bragg peaks with the peaks observed in X-ray diffraction analysis of untreated dentin and dentin irradiated with Nd:YAG laser. More detailed X-ray patterns of two untreated and two irradiated dentin samples are exposed in Figure 4. Untreated dentin showed diffraction peaks that match with the hydroxyapatite Bragg peaks. However, laser irradiated dentin presented some new additional Bragg peaks when compared to untreated dentin (blue arrows), close to the diffraction peaks of the expected pattern for TetCP. No changes on X-ray diffraction patterns of dentin were observed due to APF application when compared to untreated dentin; as well, the application of APF before Nd:YAG laser irradiation did not promote additional changes on the diffraction patterns of dentin when compared to Nd:YAG laser irradiation alone.

**Figure 5:**
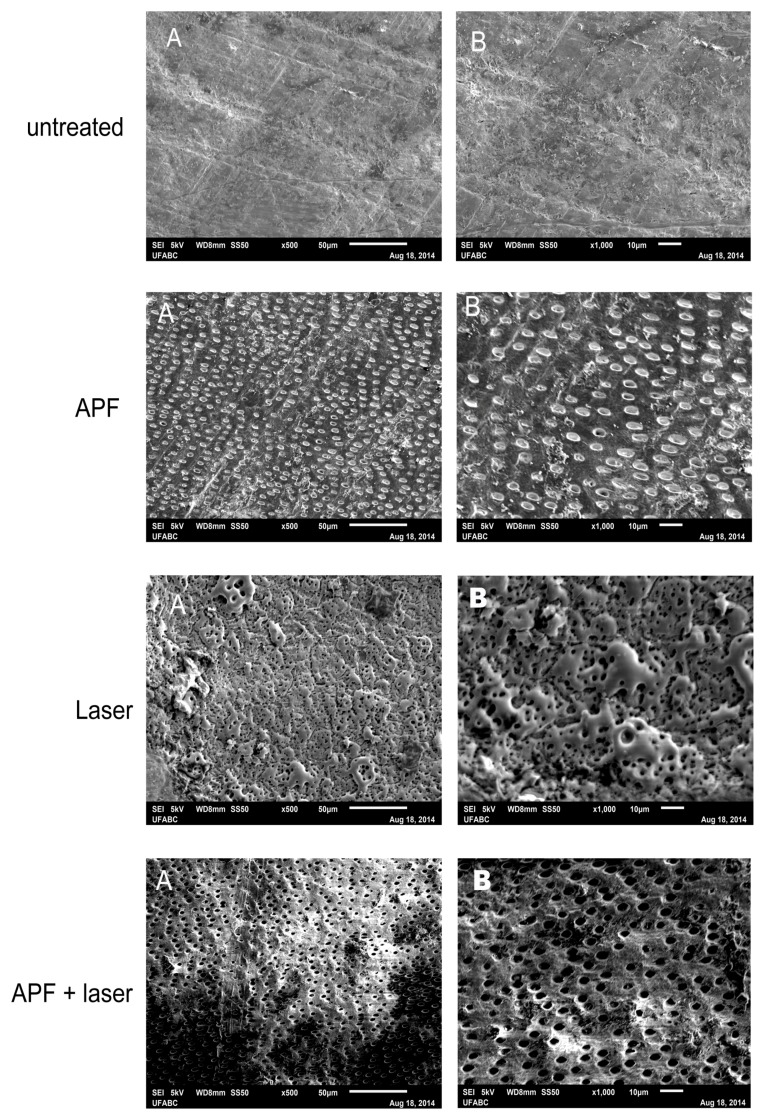
Representative scanning electron microscopy (SEM) images of dentin surfaces of each experimental group, immediately after treatments. Original magnification: (**A**) 500×; (**B**) 1000×.

The morphological changes promoted by treatments are observed in Figure 5. On untreated slabs there is a flat surface, which is covered by a smear layer and without the exposition of dentin tubules. The APF application promoted the removal of this smear layer, the exposition of the opened dentin tubules, and the formation of some globules among dentin tubules. Root dentin irradiated with Nd:YAG laser showed irregularities and depressions, characteristics of melting of the surface, with zones of fusion and ressolidification in a mosaic pattern. The association of APF and Nd:YAG laser promoted a slight ablation of the surface, with the opening of the dentin tubules and without the formation of globules.

The area and depth of wear lesions for each experimental group after 5, 10, and 15 days of erosive/abrasive cycling are shown in Table 1 and Table 2, respectively. Evidence shows that the APF application did not promote statistically significant changes on the area and depth of wear lesions when compared to untreated slabs in any period of erosive/abrasive cycling (*p* > 0.05). However, Nd:YAG laser irradiation significantly reduced (*p* < 0.05) the area and depth of wear lesions in all analyzed days. Although with no statistically significant difference between groups (*p* > 0.05), APF application before laser irradiation appears to have increased the area (Table 1) and depth (Table 2) of erosion lesions when compared to the only irradiated group, which suggests that APF hampers the effect of the laser irradiation.

**Figure 6:**
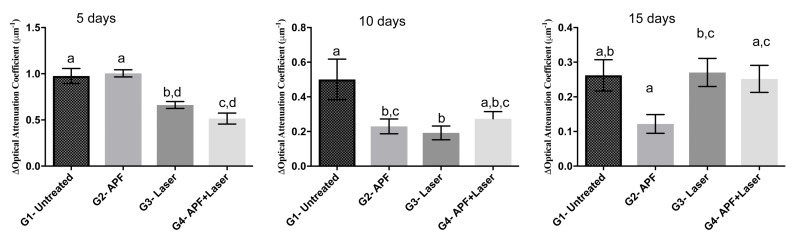
Average of optical attenuation coefficient of all experimental groups 5 days, 10 days, and 15 days after submission to an abrasive/erosive cycling. Bars denote standard error. Distinct letters indicate statistically significant differences (*p* < 0.05) according to Tukey’s test.

Figure 6 shows the average of optical attenuation coefficient obtained for all experimental groups of this study, in different periods of abrasive/erosive cycling. It is observed that after 5 days the optical attenuation coefficients of irradiated groups were significantly lower (*p* < 0.05) than the untreated or samples treated with APF-gel. After 10 days of erosive cycling, all treated samples presented optical attenuation coefficient lesser than the untreated ones, with exception for the group that had the association of treatments. After 15 days, it was noted that none of the treatments had optical attenuation coefficient different than the untreated group; however, the lased group had optical attenuation coefficient significantly higher than the APF-gel treated group, and the application of APF before laser promoted a slight decrease on optical attenuation coefficient when compared to samples that were only laser irradiated.

## 3. Discussion

Although infrared high-intensity laser irradiation is a widely suggested alternative for preventing enamel caries, there are few studies that relate the preventive effects for erosion lesions [22,23,24,25,26]. Also, the results of these studies are controversial, since different laser wavelengths and erosive conditions were evaluated. The literature report also the use of Nd:YAG laser for preventing or treating enamel [13] and dentin [16,17,18,19,20,21] erosion; however, the evaluations were performed using different laser parameters, different associations with therapeutic agents, and using different erosive protocols. These laser parameters adjusted in the present study were used because they were enough to promote the melting of the dentin surface, as can be seen in Figure 5; additionally, they were shown to be safe for pulp vitality when used with the coal paste as the photoabsorber [5,27]. The melting of the surface indicates that sufficient temperatures have been reached to promote chemical changes in the dentin, as shown in Figure 3 and Figure 4. Also, the contradictory results can be due to the absence of association with a photoabsorber in most of the literature studies, with aims to restrict the absorption of photons on the surface of enamel and dentin. The use of a photoabsorber is strictly necessary when using Nd:YAG laser for increasing the temperature of dental hard tissues, taking into account the fact that photons with wavelength of 1064 nm have low absorption by water and hydroxyapatite [7], which are the main components of enamel and dentin.

According to the literature [28,29], the increase in surface temperature is the responsible for the structural changes that occur after laser irradiation. In fact, the present study showed that Nd:YAG laser irradiation significantly reduced the proportion of carbonate of root dentin when compared to the phosphate amount, indicating the occurrence of carbonate loss due to laser irradiation. The carbonate radical can substitute the hydroxyl or phosphate radicals in the biological apatite crystal, forming a less stable and less soluble phase [28]; in this way, the reduction of carbonate content is related to the increase of the resistance of enamel to demineralization. A previous study from our group corroborates this finding when using Nd:YAG on enamel [29], and this fact indicates that the temperature rise during laser irradiation was higher than 400 °C [30,31]. Decomposition of the organic matrix (above 350 °C) has also been reported, but this was not detected in our FTIR study.

However, the APF treatment alone or associated with laser irradiation promoted a significant increase on the organic matrix proportion, which can be due to the thickener used in the formulation of APF-gel. In Figure 1B, there is an expressive amount of amide I and II on APF-gel spectrum, and these components may be retained on the sample even after washing them, overlapping the absorption bands of the dentin. The application of APF-gel prevents dental demineralization mainly by forming a 0.2–0.4 µm thick CaF_2_-like material layer on the surface [32], which presents absorption bands at 3400, 1550, and 364 cm^−1^, which corresponds to H–O–H bending of H_2_O molecules and rotations of the hydroxyl ions, respectively [33]. In this study, it was not possible to detect the presence of CaF_2_-like material layer by FTIR spectroscopy, because these absorption bands are overlapped with the main infrared dentin bands, which limits the use of this technique for this purpose.

Considering the crystalline changes promoted by treatments, our X-ray diffraction analysis demonstrated that irradiation of root dentin with Nd:YAG laser induces the formation of one minor crystalline phase mixed in the crystalline hydroxyapatite [Ca_5_(PO_4_)_3_OH], whose some peaks match with tetracalcium phosphate (TetCP). This result suggests that the Nd:YAG irradiated dentin is composed of a mixture of crystalline phases, in which hydroxyapatite prevails but there are traces of TetCP. However, it was not possible to precisely determine the lattice parameters corresponding to the hexagonal unit cell of the TetCP phase, taking into account the overlapping of diffraction peaks of the TetCP phase with the hydroxyapatite. Similar results were observed on enamel irradiated with Ho:YLF, Nd:YAG, and Er,Cr:YSGG lasers [34,35,36], in which some minor phases were found after laser irradiation. These results indicate that temperatures above 1100 °C were reached during irradiation, since the literature reports that the formation of α and β-tricalcium phosphates, as well as tetracalcium phosphate and bruxite, occurs above this temperature [30,31]. As expected, the application of APF-gel does not result in any crystalline changes on dentin, and it was not possible to detect the CaF_2_-like material layer signal considering that it is a very thin and non-homogeneous layer.

The chemical effect promoted by Nd:YAG laser on root dentin is expected, since our SEM observations showed melting and recrystallization of root dentin, which indicates the temperature of dentin surface increased up to 1100 °C [30]. The same morphological findings were reported on enamel, using the same irradiation conditions [27] and also on dentin under different energy densities [37]. The presence of melting and recrystallization of the surface apparently has an important effect on the diffusion of ions during demineralization process, because melted layer can act as a sealing in this process [38]. However, considering the efficacy that other laser wavelengths presented on reducing dental caries even without promoting these morphological effects [6], and also the chemical effects promoted due to the temperature rises, this is the main reason for reducing diffusion of hydrogen, calcium, and phosphate through the inter-crystalline and inter-prismatic spaces during the demineralization process.

Our study agrees with this hypothesis, considering that laser irradiation on root dentin significantly reduced the area and depth of erosion/abrasion lesions observed after 5, 10, and 15 days of erosive/abrasive challenge. Taking into account the erosive process, the literature presents controversial results [16,17,18,19,20,21], mainly explained by different erosion protocols, different laser parameters, and the association (or not) with a photoabsorber. In this study, a longer erosion/abrasion protocol was used, which simulates a clinical condition of an erosive attack, abrasion with a toothbrush, and remineralization with fluoridated saliva, which gave rise to the action of both fluoride from APF-gel and laser irradiation. However, in all considered periods, it was shown that the CaF_2_-like material layer formed after APF-gel application was not enough to prevent dentin wear, while the dentin modification due to laser irradiation seemed to be more efficient. It must be pointed out that CaF_2_-like material formed due to APF-gel application may have been released at the solutions used at the erosive/abrasive cycling, considering that the CaF_2_-like material concentration decreases 10 times after 48 h in the continuous flow of artificial saliva [39]. In this way, the APF-gel effect was restricted to earlier periods of demineralization.

In the present work, the application of APF-gel before Nd:YAG laser did not decrease the depth and area of wear lesions when compared to the isolated effect of laser irradiation, but it seemed that the APF-gel hampered the effects of laser irradiation, probably decreasing the absorption of photons and, as a consequence, decreasing the heating effect. Also, the obtained results suggest that high-intensity laser irradiation presents a long-lasting effect when compared to the effects promoted by APF-gel application, and the association of treatments had no benefits when compared to the effects of treatments alone. The literature is contradictory on reporting the effects of the association of laser with fluoride [40,41,42,43], and it depends mainly if APF-gel is applied before or after laser irradiation. While some studies state that laser can attenuate or even increase fluoride diffusion into dental hard tissue [42], others say that laser irradiation can favor the CaF_2_-like material formation and retention on the irradiated surface [6,44]. For that effect, APF-gel should be applied after laser irradiation, which was not performed in the present study. The reason for applying the APF-gel before the laser irradiation would be to verify the possibility of greater retention of the CaF_2_-like material in the dentin by the thermal effect, which does not seem to have been of any benefit when compared to the effects of the treatments alone.

The value of the attenuation coefficient is an optical measurement obtained by OCT technique that is related to the identification of sound and demineralized tissues, as already shown in literature [45,46,47,48]. In this study, it was noticed that the values of optical attenuation coefficient of all samples decreased during the erosive/abrasive cycling days. This finding agrees with some previous reports [46,48] that indicate that the demineralization promoted by acids, such as the citric acid contained in the soft drink used in this study, creates empty spaces in the structure of dentin that increases the number of interfaces and, as a consequence, increases the scattering of light and decreases the values of optical attenuation coefficients. However, there are some studies [45,47] that relate that the values of optical attenuation coefficient increase in demineralized samples considering that the mineral loss creates spaces in the structure of tissues that propitiate the higher penetration of light and, in this way, it decreases the scattering of light. It must be considered that the above-mentioned studies were performed on enamel, whose structure differs from dentin, and comparison between such different tissues is difficult.

Laser-irradiated samples presented a significant decrease in the optical attenuation coefficient after 5 and 10 days of abrasive/erosive cycling, and we can infer that laser promoted a preventive effect reducing the diffusion of acids within the dentin. However, after 15 days of erosive/abrasive cycling, no statistical difference was observed when comparing irradiated samples with the untreated ones. This occurred, probably, because the abrasion by toothbrush tends to remove the softened tissue by the erosion process, so that the dentin analyzed after 15 days of cycling is healthy and attenuates more the light than the dentin present in 5 days of cycling. Also, the absence of a statistically significant difference in the comparison between the irradiated samples and the irradiated and fluoride-treated samples indicates that the association of treatments is not more efficient than the laser irradiation alone.

In this way, according to the results obtained in this study, it was possible to conclude that Nd:YAG laser irradiation, even at the low energy density used in the present study but in association with a photoabsorber, alters the microstructure of root dentin, and these changes are the responsible for the reduction on the progression of erosion and abrasion lesions. Also, the irradiation after topical fluoride application does not offer any additional benefit on wear prevention.

## 4. Materials and Methods

### 4.1. Experimental Design

In a blind in vitro study, 180 bovine root dentin slabs were randomly distributed into four experimental groups of 45 samples in each group: G1—untreated, G2—treated with acidulated phosphate fluoride gel (APF-gel, 1.23% F^−^) for 4 min, G3—irradiated with Nd:YAG laser at 84.9 J/cm^2^ [5,27], and G4—APF-gel application followed by Nd:YAG laser irradiation. The compositional, crystalline, and morphological effects promoted by all treatments were evaluated by Fourier transformed infrared spectroscopy (FTIR), X-ray diffraction (DRX), and scanning electron microscopy (SEM), respectively, on five samples of each experimental group immediately after treatments. The remaining 45 samples of each experimental group were again randomized in 3 groups of 15 specimens each, in which samples were submitted to an erosive and abrasive demineralization (using Sprite Zero, pH = 2.8, 90 s, 4 times a day), and remineralisation (with artificial saliva, pH = 7.4) cycling [16] for 5, 10, and 15 days, respectively. During the erosive/abrasive cycling, twice a day, all slabs were brushed for 15 s with a load of 1.5 N using a 0 ppm F^−^ dentifrice [49]. The area (µm^2^) and depth (µm) of erosion/abrasion lesions were evaluated by optical coherence tomography (OCT) at the initial and at the end of erosive/abrasive cycling process. Also, the optical attenuation coefficient of all samples was evaluated. An individual statistical analysis for each variable response (composition, area, depth, and optical attenuation coefficient) was performed at the level of significance of 5%. For the statistical analysis, each treatment was considered a separate block, and the experimental unit was the slab (*n* = 15).

### 4.2. Preparation of the Specimens and Treatments

After the approval from the Animal Ethics Committee of the Federal University of ABC (CEUA-UFABC No. 004/2013 approved on April 24, 2013), the root dentin slabs (4 mm × 4 mm × 2 mm) were obtained from cervical surfaces of 200 bovine incisor teeth. All samples were examined on stereomicroscope to verify the absence of cracks and irregularities; after that, the selected slabs were cleaned with pumice for 10 s, and kept in humid environment under refrigeration until the beginning of the experiments.

The laser irradiation was performed using a Nd:YAG laser device (Power Laser TM ST6, Lares Research, Chico, CA, USA), which operates at wavelength of 1.064 μm and temporal width of 100 μs. The energy is delivered through a fiber optic system with 300 μm of spot size. Nd:YAG laser was used to irradiate all slabs surfaces, scanning them by hand under the conditions of 0.6 W, 60 mJ per pulse, energy density of 84.9 J/cm^2^ , and 10 Hz repetition rate [5]. Before irradiation, the energy per pulse was calibrated by an energy/power meter (FieldMaster, Coherent, Santa Clara, CA, USA) and the surfaces of the slabs were covered with a thin layer of a photosensitizer composed of triturated coal diluted in equal parts of deionized water and 99% ethanol [27].

The acidulated phosphate fluoride gel (1.23% F^−^, 0.1 M of phosphoric acid, pH 3.6 to 3.9—Flutop Gel^®^, SSWhite, Rio de Janeiro, Brazil) was applied to the dentin slabs with a cotton swab for 4 min, followed by washing with distilled and deionized water during 1 min and dried with absorbent paper [41]. After treatments, all surfaces of specimens were protected with two layers of an acid-resistant varnish, except for an area of 8 mm^2^ of dentin, on which the treatments were applied. The objective of this procedure was to maintain a reference surface for wear determination after erosive/abrasive cycling.

### 4.3. Compositional Analysis

Five samples of each experimental group, which were not subjected to the erosive/abrasive regimen, were cleaned on ultrasonic bath for 1 min and analyzed using the attenuated total reflectance technique of the Fourier transform infrared spectroscopy (ATR-FTIR, FTIR 660—Varian IncA., Palo Alto, CA, USA). The spectra of each sample were obtained with 4.0 cm^−1^ resolution, with 80 scans in the range of 4000 to 600 cm^−1^ [36]. In each slab, a single spectrum in an area of 1.5 mm^2^ was collected, which corresponded to the size of diamond crystal.

The absorption bands considered for this study were phosphate (1300–900 cm^−1^), the ν_2_ vibration mode of carbonate (around 870 cm^−1^), the superposition of the stretching ν_3_, and bending ν_4_ vibration mode of carbonate (1600–1300 cm^−1^), as well as the bands that correspond to the organic matrix of dentin-amide I (1680–1600 cm^−1^), amide II (1580–1480 cm^−1^), and amide III (1200–1300 cm^−1^). For semi-quantification, the areas under the considered bands were calculated after tracing the baseline, and normalization was done by subtracting the area of the considered bands by the area of the phosphate band [36].

### 4.4. Crystallographic Analysis

The crystalline structure of the same samples evaluated by ATR-FTIR spectroscopy was assessed by X-ray diffraction (XRD) at a synchrotron XRD1 beamline (LNLS, Campinas, Brazil), using a monochromatic X-ray beam with wavelength of 0.0954 nm. The X-ray beam was configured at a grazing angle in order to maximize the surface diffraction signal and better detect the possible new crystallographic phase produced after the laser irradiation. A step scanning diffractometer (2θ step = 0.01°) equipped with a scintillator photon counter was used for recording the diffraction spectra. In order to reduce the effect of statistical errors, a single diffraction pattern was obtained as the sum of the two individual recorded patterns [34,35,36].

The procedure for indexing the peaks that were observed during the XRD analysis was started by using the database PDF2—Release (International Centre for Diffraction Data—ICDD, 2005). The determination of the lattice parameters was performed as described before [34,35,36].

### 4.5. Morphological Analysis

The same samples analyzed before were fixed with 2% glutaraldehyde solution for 2 h and were immediately immerged in a phosphate-buffered solution 0.1 M at room temperature, rinsed with distilled water, and then dehydrated in a graded series of alcohol solutions (50%, 70%, 80%, 90%, 95%, and 100%) for 10 min at each concentration. These samples were sputtered with a 15 µm thick gold and were attached to aluminum stubs with a self-adhesive carbon conductive tape for analysis [6,27]. Samples were examined by a scanning electron microscope (JEOL JSM-6010LA, Tokyo, Japan), under magnifications of 500× and 1000×.

### 4.6. Erosive/Abrasive Cycling

Forty-five samples of each experimental group were submitted to an erosive/abrasive de- and remineralization cycling, according to Magalhães et al. (2010) [16] protocol, which lasted for 5 (15 samples/group), 10 (15 samples/group), or 15 days (15 samples/group) according to the described before. The erosion process was performed with the immersion of each sample individually in 30 mL of freshly opened bottle of Sprite Zero^®^ drink (Coca-Cola Company, Rio de Janeiro, Brazil, pH 2.6, unstirred, 25 °C) four times daily for 90 s each. After each erosive challenge, the samples were washed with distilled water for 5 s, dried with absorbent paper, and individually immerged in 30 mL of the remineralization solution (1.5 mM Ca(NO_3_).4H_2_O, 0.9 mM NaH_2_PO_4_·2H_2_O, 150 mM KCl, 0.03 ppm F^−^, TRIS buffer, pH 7.0) [50]. To simulate the abrasion process by toothbrushing, twice a day the samples were individually exposed to 25 mL of freshly made slurries of a non-fluoridated toothpaste (1 toothpaste:3 water, Malvatrikids Baby, Daudt, Rio de Janeiro, Brazil) [49] and then abraded using an electrical toothbrush for 10 s (166 oscillations/s, Pro-saude Power, Oral-B, Sao Paulo, Brazil) [16]. The force, duration, and position of toothbrush on each sample were standardized according to literature studies [51,52] in order to simulate a clinical condition.

### 4.7. Wear Depth and Area Assessment

The measurement of wear depth and area after the erosive/abrasive cycling, as well as the optical attenuation coefficient, was performed using an optical coherence tomography system (OCT—OCP930SR, Thorlabs Inc., Newton, NJ, USA), with 2 mW of optical power, central wavelength of 930 nm, spectral bandwidth of 100 nm, axial resolution of 4.0 µm, and lateral resolution of 6.0 µm. For OCT measurement, the nail varnish of the surface of samples was carefully removed using a scalpel. The tomographic images were obtained from 5 previously demarcated scanning lines of each slab (positioned at intervals of 0.5 mm), which started from the first reference surface across the exposed area and finished on the other reference area. The positioning of each scanning line was standardized and maintained during all the experiments. All samples were examined 2 times: immediately after treatments and after the erosive/abrasive cycling period defined for each group.

The obtained tomographic images (B-scan) were analyzed using the ImageJ software (National Institute of Health, Bethesda, MD, USA), which allows the user to define a region of interest, as well as the area and depth of lesions. The measurement of area and depth of the wear lesions was performed considering four equidistant points of each image, and the average of the four points of the five scanning lines was performed for each slab.

The mean of optical attenuation coefficient of each B-scan was analyzed using an algorithm developed in LabView 8 (National Instruments, Austin, TX, USA), in which was used an equation similar to Beer-Lambert law to calculate the optical attenuation coefficient considering the intensity of backscattered light as a function of depth in sample. The procedure of this calculation was described before [46].

### 4.8. Statistical Analysis

The data of compositional analysis, the area and depth of lesions, and the optical attenuation coefficient were statistically analyzed. Before the statistical analysis, the independence, homogeneity, and normality of the variances of experimental data were tested. The independence was assured by the way the experiments were conducted. The homogeneity was evaluated by Levene’s test and the normality was evaluated by Shapiro-Wilk’s test (all tests were performed at a significance level of 5%). After the confirmation of all these requirements, the composition data was statistically analyzed by Kruskal-Wallis and Student Newman Keuls; additionally, the area, depth, and optical attenuation coefficient were analyzed by ANOVA (two-factor with replication) followed by Tukey’s test. For all analyses, 5% was considered the limit of significance, and the software SPSS 13.0 for windows (SPSS Inc., Chicago, IL, USA) was used.

## Figures and Tables

**Table 1:** Means ± standard deviation (µm^2^) of area of dentin wear according to the treatments and duration of erosive/abrasive cycling. Treatments followed by distinct superscript letters differ statistically by the Tukey test (*p* < 0.05).

Groups	5 Days (*n* = 15)	10 Days (*n* = 15)	15 Days (*n* = 15)
Untreated (*n* = 45)	26,344.8 ^a^ ± 4263.1	28,197.1 ^a^ ± 6470.0	34,111.5 ^a^ ± 9269.9
APF (*n* = 45)	25,882.2 ^a^ ± 3621.1	27,958.3 ^a^ ± 5318.8	35,187.2 ^a,b^ ± 8762.8
Laser (*n* = 45)	12,960.1 ^b^ ± 5162.7	15,007.1 ^b^ ± 8831.0	14,760.2 ^c^ ± 11,515.5
APF + Laser (*n* = 45)	16,171.8 ^b^ ± 5445.1	21,705.0 ^b^ ± 4446.6	24,580.1 ^b,c^ ± 9505.8

**Table 2:** Means ± SD (µm) of depth of dentin wear according to the treatments and duration of erosive/abrasive cycling. Treatments followed by distinct superscript letters differ statistically by the Tukey test (*p* < 0.05).

Groups	5 Days (*n* = 15)	10 Days (*n* = 15)	15 Days (*n* = 15)
G1—Untreated (*n* = 45)	9.78 ^a^ ± 2.9	10.66 ^a^ ± 3.6	12.80 ^a^ ± 5.0
G2—APF (*n* = 45)	9.25 ^a^ ± 2.5	10.09 ^a^ ± 1.9	14.80 ^a^ ± 3.9
G3—Laser (*n* = 45)	5.99 ^b^ ± 2.7	7.23 ^b^ ± 2.9	6.97 ^b^ ± 4.0
G4—APF + Laser (*n* = 45)	6.05 ^b^ ± 3.2	7.57 ^b^ ± 2.8	8.13 ^b^ ± 4.2
